# Is there room in epilepsy for the claustrum?

**DOI:** 10.3389/fsysb.2024.1385112

**Published:** 2024-04-03

**Authors:** Glenn D. R. Watson, Stefano Meletti, Anil K. Mahavadi, Pierre Besson, S. Kathleen Bandt, Jared B. Smith

**Affiliations:** ^1^ Department of Psychology and Neuroscience, Duke University, Durham, NC, United States; ^2^ Department of Biomedical, Metabolic and Neural Sciences, Center for Neuroscience and Neurotechnology, University of Modena and Reggio Emilia, Modena, Italy; ^3^ Department of Neurosciences, OCSAE Hospital, Modena, Italy; ^4^ Department of Neurosurgery, University of Alabama at Birmingham, Birmingham, AL, United States; ^5^ Department of Radiology, Northwestern University Feinberg School of Medicine, Chicago, IL, United States; ^6^ Department of Neurologic Surgery, Northwestern University Feinberg School of Medicine, Chicago, IL, United States; ^7^ Molecular Neurobiology Laboratory, Salk Institute for Biological Studies, La Jolla, CA, United States

**Keywords:** claustrum, claustrum sign, area tempestas, seizure generalization, impaired awareness seizures, epilepsy, status epilepticus

## Abstract

The function of the claustrum and its role in neurological disorders remains a subject of interest in the field of neurology. Given the claustrum’s susceptibility to seizure-induced damage, there is speculation that it could serve as a node in a dysfunctional epileptic network. This perspective article aims to address a pivotal question: Does the claustrum play a role in epilepsy? Building upon existing literature, we propose the following hypotheses for the involvement of the claustrum in epilepsy: (1) Bilateral T2/FLAIR magnetic resonance imaging (MRI) hyperintensity of the claustrum after status epilepticus represents a radiological phenomenon that signifies inflammation-related epileptogenesis; (2) The ventral claustrum is synonymous with a brain area known as ‘area tempestas,’ an established epileptogenic center; (3) The ventral subsector of the claustrum facilitates seizure generalization/propagation through its connections with limbic and motor-related brain structures; (4) Disruption of claustrum connections during seizures might contribute to the loss of consciousness observed in impaired awareness seizures; (5) Targeting the claustrum therapeutically could be advantageous in seizures that arise from limbic foci. Together, evidence from both clinical case reports and animal studies identify a significant role for the ventral claustrum in the generation, propagation, and intractable nature of seizures in a subset of epilepsy syndromes.

## 1 Introduction

In epilepsy, a disorder characterized by recurring, spontaneous seizures, the challenge of diverse etiologies complicates both seizure localization and treatment decisions (Watson et al., 2021a). Although significant progress has been made in understanding the neural underpinnings of epilepsy by viewing it as a brain network disorder, the ability to leverage this network for therapeutic purposes remains elusive. To overcome these shortfalls and identify novel targets for epilepsy therapeutics, recent studies have delved into the molecular and epigenetic changes that occur in key brain regions associated with seizure activity, namely, the hippocampus (Conboy et al., 2021; Pires et al., 2021). But another avenue has emerged based on neuroimaging results pointing to an obscure brain region, the claustrum, which appears to have a role in aberrant networks that give rise to the hyperexcitability underlying epilepsy.

Situated beneath the insular cortex, between the external and extreme capsules, the claustrum is a distinctive subcortical structure whose geometry can be described as a thin sheet of glutamatergic projection neurons, extending across the anteroposterior extent of the forebrain. The claustrum’s connectivity is extensive, innervating the entire cerebral cortex, including the contralateral cortex, as well as receiving inputs from both hemispheres (for review see Smith et al., 2019a). These connections are topographically organized based on modality across the claustrum’s dorsoventral extent, with limbic connections concentrated in its ventral portion (Smith and Alloway, 2014; Watson et al., 2017; Marriott et al., 2021). In fact, renewed interest in the role of the claustrum in epilepsy stems from these limbic connections with brain regions frequently identified as seizure foci, such as the mediodorsal thalamus, hippocampus, and amygdala (Jackson et al., 2020; Smith et al., 2020; Benarroch, 2021).

Clinically, claustrum lesions that cause seizures often encompass surrounding structures, complicating the identification of the claustrum’s specific role. Recent structural magnetic resonance imaging (MRI) studies have identified a distinctive signature that prominently features the claustrum in patients with intractable seizures, providing compelling evidence of its involvement in epilepsy. To support this perspective, we begin by reviewing case study evidence of radiological signals in the claustrum of new-onset seizure patients with acute encephalopathies. Subsequently, we turn to rodent models of epilepsy to precisely define the claustrum’s involvement, allowing us to distinguish subregions implicated in seizure generation and propagation, particularly its ventral portion known to have significant connections with the limbic system (Watson et al., 2017). The culmination of these separate approaches has led us to the novel hypothesis that the ventral claustrum is in fact synonymous with the so called ‘area tempestas,’ a non-circumscribed brain area traditionally implicated in seizure propagation and epileptogenesis. We also speculate that disruption of claustrum connections with the thalamus and cortex may impair consciousness during certain seizure subtypes. With this evidence, we conclude by exploring therapeutic development centered on the claustrum and identifying the indications most likely to benefit from claustrum remediation.

## 2 Misleading signs? The claustrum’s role in *de novo* status epilepticus

**FIGURE 1:**
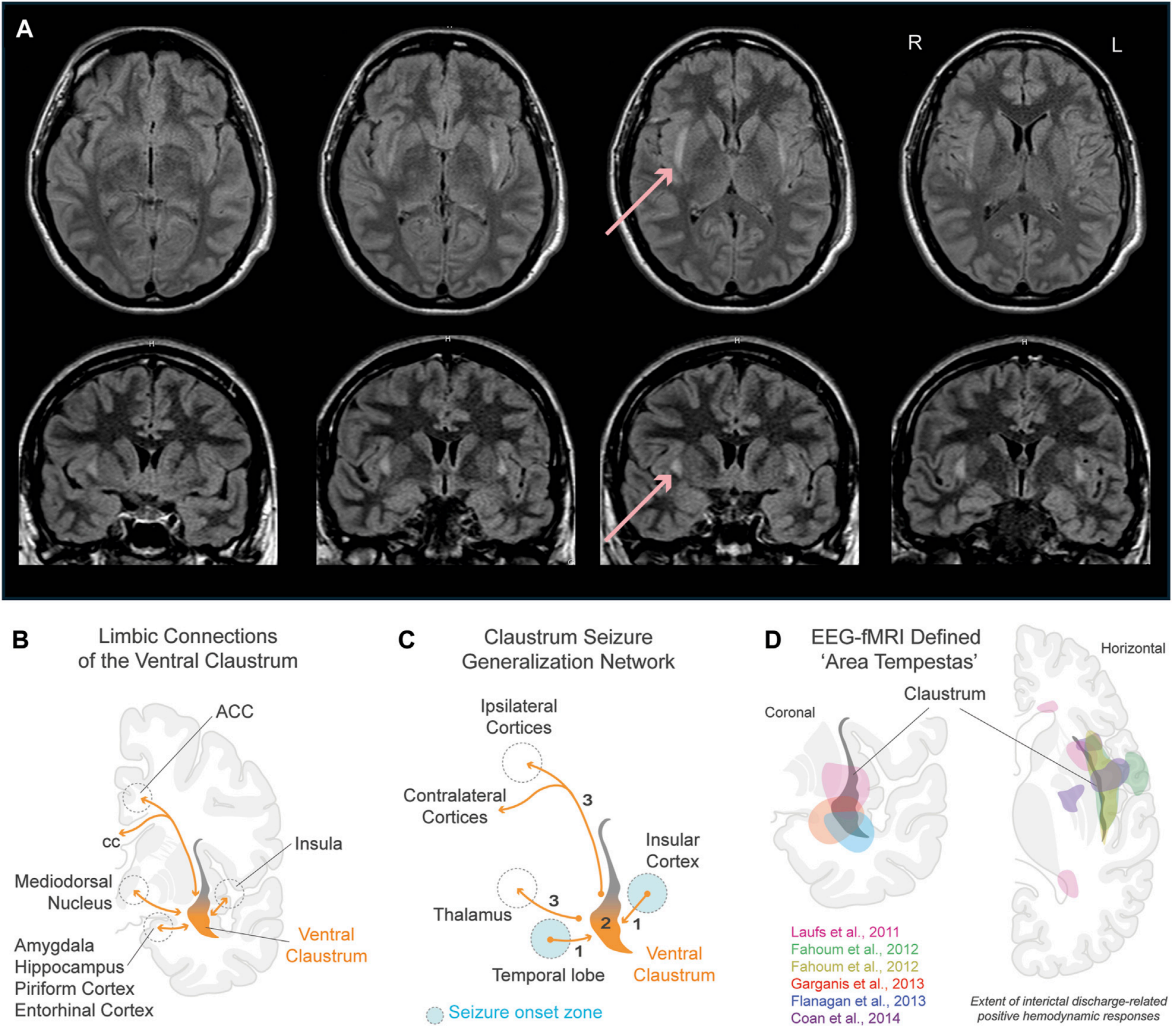
Role of the claustrum in seizures. **(A)** Claustrum sign in a 24 year old female 7 days from onset of status epilepticus after febrile illness refractory to antiseizure medications (Meletti et al., 2015). Horizontal (top row) and coronal (bottom row) sections show extent of bilateral claustrum FLAIR MRI hyperintensity. Red arrows point to claustrum hyperintensity in one section. **(B)** Limbic connections of the ventral claustrum. Reciprocal connections (orange lines) are shown with the hippocampal system, basolateral amygdala, piriform cortex, entorhinal cortex, insular cortex, medial PFC, and ACC. **(C)** Claustrum seizure generalization network. Schematic illustrates the claustrum as a secondary node propagating seizures arising from limbic brain structures to ipsi- and contralateral motor-related cortices. **(D)** Physiologically defined ‘area tempestas’ in epilepsy patients using EEG-fMRI. Overlayed colored areas correspond to approximated areas of interictal discharge-related positive hemodynamic responses in studies color coded within panel. Pink: Laufs et al., 2011, group EEG-fMRI analysis for a mixed cohort of focal epilepsy patients (n = 19). Green and yellow: Fahoum et al., 2012, group EEG-fMRI analysis results of temporal lobe epilepsy group (n = 32) for hemodynamic response functions peaking at 3s (green) and 5s (yellow) after interictal epileptic discharges. Red: Garganis et al., 2013, discharge-correlated BOLD change in a patient experiencing recurrent focal seizures following temporal lobectomy. Blue: Flanagan et al., 2013, group EEG-fMRI random effects analysis for a mixed epilepsy cohort (n = 27). Purple: Coan et al., 2014, group EEG-fMRI T-maps from mesial temporal lobe epilepsy patients with hippocampal sclerosis (n = 13). Refer to studies for original EEG-fMRI overlays t-score values, and *p*-values. Abbreviations: ACC, anterior cingulate cortex; BOLD, blood-oxygen-level-dependent; cc, corpus callosum; EEG, electroencephalogram; fMRI, functional magnetic resonance imaging; FLAIR, fluid-attenuated inversion recovery; PFC, prefrontal cortex.

Case reports of patients with status epilepticus (SE) have provided valuable insights into the involvement of the claustrum in seizures (Meletti et al., 2015; Meletti et al., 2017; Atilgan et al., 2022). In the acute phase of SE, a distinct hyperintensity localized to the claustrum emerges in T2-weighted-fluid-attenuated inversion recovery (T2/FLAIR) MRI images latent from seizure onset, as illustrated in Figure 1A. This radiological occurrence, termed ‘claustrum sign,’ is notable for its association with generalized tonic-clonic seizures and its reversibility following SE resolution (Table 1). Intriguingly, claustrum-related imaging abnormalities are rare in patients with SE but are strongly linked to a *de novo* SE that typically develops in young, healthy patients that are refractory to antiseizure medications. In some cases, autoimmune antibody positive encephalitic syndromes have been reported, including some cases of COVID-19 post-infection encephalitis (Ayatollahi et al., 2021; Humayun et al., 2023). However, the etiology in most cases remains undetermined with patients being described in the context of febrile infection-related epilepsy syndrome (FIRES) and new-onset refractory status epilepticus (NORSE) (see updated terminology in Hirsch et al., 2018). Thus, the claustrum sign serves as a distinctive radiological biomarker, suggesting a potential link to inflammation-related epileptogenesis and cytokine-mediated neuroinflammation. But does this hyperintensity indicate a *causative* role for the claustrum in inflammation-related SE, or does the claustrum sign simply signify inflammation?

**TABLE 1:** Appearance of Claustrum Sign in Cases of Generalized Tonic-Clonic Seizures and Status Epilepticus.

Case Study	Patient	Seizure Type	EEG	MRI Findings	Diagnosis
Ayatollahi et al. (2021) (North America)	18 y/o F	GTCS	Theta/delta slow-wave activity	Unremarkable day 7, bilateral claustrum T2/FLAIR hyperintensity day 21, near-complete resolution one month after	COVID-19 post-infectious encephalitis
Di Dier et al. (2023) (Europe)	39 y/o F	Focal evolving into SE	N/A	Bilateral claustrum T2/FLAIR hyperintensity	FIRES
Guo and Hong (2023) (Asia)	19 y/o F	SE	Bilateral multifocal discharges, left hemisphere predominance	Bilateral claustrum T2/FLAIR hyperintensity day 19	FIRES
Humayun et al. (2023) (Asia)	6 y/o F	GTCS	Moderate amplitude 4 Hz theta, intermixed delta	Bilateral claustrum T2/FLAIR hyperintensity	COVID-19 post-infectious encephalitis
Hwang et al. (2014) (Asia)	28 y/o F	SE	Generalized spike and waves at 1-1.5 Hz	Bilateral claustrum T2/FLAIR hyperintensity day 27†	SE with unknown etiology
Ishii et al. (2011) (Asia)	21 y/o M	GTCS evolving into SE	Slow basic rhythms with epileptic discharges	Unremarkable day 7, bilateral claustrum T2/FLAIR hyperintensity day 13, resolution day 26	Mumps encephalitis
Muccioli et al. (2022) (Europe)	40 y/o F	SE	Bilateral asymmetric lateralized periodic discharges, predominance in right fronto-temporal region	Bilateral claustrum T2/FLAIR hyperintensity	FIRES
Nixon et al. (2001)* (Europe)	35 y/o M	GTCS evolving into SE	Generalized slow wave	Unremarkable day 7, bilateral claustrum T2/FLAIR hyperintensity day 13 (4 days after SE onset)	SE with unknown etiology
Safan et al. (2023) (Asia)	30 y/o M	GTCS evolving into SE	Continuous left-sided epileptiform discharges, left middle temporal predominance	Bilateral external/extreme capsule hyperintensities with bilateral claustrum sparing day 9, resolution day 37	Seronegative limbic encephalitis
Silva et al. (2018) (Europe)	6 y/o F	SE	Occipital intermittent rhythmic delta activity	Bilateral external/extreme capsule hyperintensities day 22, reduction at month 3	NORSE
Silva and Sousa (2019) (South America)	16 y/o F	SE	N/A	Bilateral claustrum T2/FLAIR hyperintensity day 21†, resolution 4 months later	N/A
Sperner et al. (1996) (Europe)	12 y/o F	SE followed by focal impaired awareness	Severe generalized slowing, right-sided sharp slow waves	Bilateral claustrum T2/FLAIR hyperintensity and T1 hypointensity day 21, resolution day 25, normal MRI at week 7	SE with unknown etiology

Dates of MRI findings extrapolated from first instance of symptoms reported within case studies. See articles for list of negative laboratory findings. Diagnoses are listed as presented in case studies. Note that all cases show T2/FLAIR hyperintensity restricted to the claustrum with diffusion into the external and extreme capsules without involvement of other brain regions. See Meletti et al., 2015; Meletti et al., 2017 for cohort population reports in patients with FIRES and NORSE. See Atilgan et al., 2022 for additional case studies involving hyperintensities in other brain regions appearing with claustrum sign. Abbreviations: EEG, electroencephalogram; F, female; FIRES, febrile infection-related epilepsy syndrome; FLAIR, fluid-attenuated inversion recover; GTCS, generalized tonic-clonic seizures; M, male; MRI, magnetic resonance imaging; NORSE, new-onset refractory status epilepticus; SE, status epilepticus. *Case resulted in death. † Timeframe approximated based on article text.

Unlike claustrum damage resulting from a hemorrhagic stroke or penetrating head injury, which infrequently leads to seizures, viral and autoimmune encephalitic etiologies can manifest claustrum sign (Table 1; see Atilgan et al., 2022, for additional case summaries). Two hypotheses may explain the appearance of this radiological phenomenon. The first involves postinfection neuronal loss, encompassing gliosis, spongiform degeneration, and demyelination during the recovery phase (Kimura et al., 1994; Sperner et al., 1996; Nomoto et al., 2007; Ishii et al., 2011). Lending credence to this hypothesis, the presence of ischemic cell changes and acute astrocytic reaction (astrogliosis) were observed in the claustra during histopathological analysis of a patient’s brain after a fatal SE case (refer to Table 1; Nixon et al., 2001). Conversely, a comprehensive neuropathological study found no abnormalities in the claustra of patients with chronic epilepsy and SE (Margerison and Corsellis, 1966). Another hypothesized mechanism is focal edema, gaining support from recent case studies of claustral edema in the context of refractory SE following consumption of Sugihiratkae mushrooms (Kuwabara et al., 2005; Nishizawa, 2005; Nomoto et al., 2007). Edema localized to the claustrum may therefore contribute to an aspect of the refractory nature of SE.

The claustrum sign may not solely be a structural abnormality but could instead signify network dysfunction, although it is seldom observed outside of encephalopathies (Steriade et al., 2017; Silva et al., 2018; Altigan et al., 2022). This prompts an exploration into whether viral-induced connectional changes are causative factors behind the appearance of this hyperintensity. A clue may reside in the claustrum’s strikingly high density of inhibitory kappa-opioid receptors (KORs) compared to other subcortical brain regions (Peckys and Landwehrmeyer, 1999; Stiefel et al., 2014; Cahill et al., 2022). The potential link between viral-induced KOR dysfunction and the claustrum sign, potentially driven by runaway excitation due to reduced dynorphin expression, necessitates careful consideration (Solbrig and Koob, 2004; Solbrig et al., 2006; Silva et al., 2018). More intriguingly, most case studies reporting claustrum sign in the literature originate from Asia and Europe, raising questions about the veracity of this signal’s physiological significance, or whether it represents an underreported, time-dependent radiological phenomenon appearing around one to three weeks from symptom onset (Table 1).

## 3 Gene expression changes in claustrum during seizures

The hypothesis that MRI hyperintensities may indicate aberrant hyperactivity within nodes of an epileptic network has been proposed (Silva et al., 2018; Ayatollahi et al., 2021). Examining the claustrum’s role as a node within a limbic epileptic network is a potential avenue to clarify its relationship with seizure activity. As mentioned previously, the ventral most region of the claustrum connects to various limbic brain structures that are implicated in seizure generation and epileptic pathology (Smith et al., 2020). These regions include the piriform, medial prefrontal, orbitofrontal, and entorhinal cortices, the amygdala (basolateral, central, and medial nuclei), and the anterior and mediodorsal nuclei of the thalamus (Fernandez-Miranda et al., 2008; Watson et al., 2017; Smith et al., 2019b) (Figure 1B). Despite its anatomical significance, the involvement of this limbic subsector of the claustrum has largely been overlooked in seizure research, potentially due to its obscurity and the ability to selectively modulate it without affecting neighboring white matter (Watson and Kopell, 2022).

An alternative method to delve into the claustrum’s potential involvement in seizures involves measuring its neuronal activity in validated animal models of epileptogenesis. Numerous studies investigating c-fos expression in temporal proximity to SE induced by various methods consistently show increased expression in limbic regions connected to the claustrum such as the hippocampus, piriform cortex, medial prefrontal cortex, entorhinal cortex, amygdala, and anterior nucleus of the thalamus (Morgan et al., 1987; Sitcoske O’Shea et al., 2000; Szyndler et al., 2009; Barros et al., 2015; Siow et al., 2020) (Figure 1B). Studies utilizing kainic acid, pentylenetetrazol, lithium-pilocarpine, and kindling exhibit increased c-fos expression and evidence of neuronal cell death in the claustrum itself (Willoughby et al., 1997; Zhang et al., 1997; Covolan and Mello, 2000; Sitcoske O’Shea et al., 2000; Zhang et al., 2001; Siow et al., 2020; Druga et al., 2024). In fact, a region we recently delineated as being a part of the ventral claustrum in rodents, the dorsal endopiriform nucleus, shows c-fos expression during SE only after the first convulsive seizure, corresponding to the appearance of claustrum sign after the onset of SE in humans (Table 1; Smith et al., 2020; see Majak and Moryś, 2007 for review).

In light of the emerging evidence supporting the ventral claustrum’s role in epilepsy through gene expression studies, a compelling avenue of exploration lies in understanding its potential influence on specific aspects of seizures. Building upon these insights, we next delve into a distinct aspect of claustrum involvement ‐ its potential role in impaired awareness seizures.

## 4 A case for involvement of the claustrum in impaired awareness seizures

Brain regions with changes in c-fos expression during seizures provide a biological anchor by which to interpret resting-state functional MRI (rs-fMRI) data. In one of our recent rs-fMRI studies, we observed functional connections between the claustrum and the thalamus, amygdala, and prefrontal cortex that are weakened under isoflurane anesthesia (Smith et al., 2017). Building on these results and findings in human rs-fMRI studies, we implicated the ventral claustrum as a critical node within both the salience and default-mode intrinsic connectivity networks (ICNs): interconnected brain regions that are functionally co-activated or co-deactivated during specific cognitive activities that are found to be impaired throughout epileptogenesis (Luo et al., 2011a; b; Smith et al., 2017; Smith et al., 2019b). Interestingly, a decrease in default-mode ICN activity is shown during generalized tonic-clonic seizures, and selective impairment to this ICN during seizures is associated with loss of consciousness (Danielson et al., 2011; Crone et al., 2015). This presents the possibility that ventral claustrum output could be impaired during seizures, and in turn alter ICN-mediated consciousness.

The claustrum’s role in consciousness, speculated by Crick and Koch (2005) has sparked renewed interest in research on the subject. A study on a refractory epilepsy patient undergoing stimulation mapping demonstrated that electrical stimulation near the claustrum could reversibly disrupt consciousness (Koubeissi et al., 2014). However, a later study involving several epilepsy neurosurgical patients contradicted this finding, as electrical stimulation of the claustrum did not lead to a loss of consciousness (Bickel and Parvizi, 2019). Nevertheless, we do not entirely rule out the possibility of the claustrum’s involvement in seizures that impair awareness via ICN alterations. Cases with claustrum sign often report impairment in consciousness (see Atilgan et al., 2022 for review). Furthermore, most focal impaired awareness seizures arise from the temporal lobe, where many of its structures directly project to the ventral claustrum, with more than half evolving into focal to bilateral generalized seizures (Kumar and Sharma, 2023).

An ongoing clinical trial (NCT04897776, 2024) stimulating the intralaminar thalamus to restore arousal in temporal lobe epilepsy patients with impaired conscious awareness may offer mechanistic insight. As shown in Figure 1C, seizures emanating from limbic brain structures could impair interactions between the claustrum, cortex, and thalamic nuclei. A robust and common target of both the intralaminar thalamus and the claustrum is the anterior cingulate cortex: where seizures often lead to impaired consciousness and motor manifestations, often involving the temporal lobe (Alkawadri et al., 2016; Benarroch, 2021). We previously hypothesized that the connectivity between the claustrum and cingulate cortex plays a major role in salience and default-mode ICNs (Smith et al., 2019a; Kou et al., 2023). Building upon this, we further hypothesize that disruption to this critical network connection may impair awareness during seizures originating from temporal lobe structures through its interaction with motor-related cortical areas (cingulate cortex) and the thalamus. We therefore support the viewpoint that while the claustrum can influence the consciousness “master switch” of a brainstem and diencephalic origin, it is not the master switch itself (Blumenfeld, 2014; Gummadavelli et al., 2015).

## 5 The ventral claustrum is synonymous with ‘area tempestas’: a brain region imlicated in seizure generation and propagation

It is plausible that the claustrum is a node by which seizures can generalize or propagate from limbic-connected structures to cortical regions considering the functional connectivity data discussed above. Supporting this possibility, from animal data, amygdaloid kindling studies reveal that claustrum lesions destabilize or entirely block seizure generalization (Wada and Kudo, 1997; Wada and Tsuchimochi, 1997; Mohapel et al., 2000). Interestingly, a non-circumscribed anatomical region termed ‘area tempestas’ traditionally described within the deep piriform cortex (primary olfactory) demonstrates strikingly similar kindling results (Löscher et al., 1995). Upon further research, the dorsal endopiriform (rodents) and pre-endopiriform (human) nuclei (i.e., ventral claustrum) correspond to this physiologically defined area (see Majak and Moryś, 2007; Vaughan and Jackson, 2014 for review).

Insight into the exact functional relationship amongst the ventral claustrum and limbic brain structures during seizures can be further gleaned from a formative electroencephalogram (EEG)-fMRI study involving focal epilepsy patients (Laufs et al., 2011). Regardless of the localization of interictal and ictal activity, the study identified a common, tightly localized brain region attributed to be the “human equivalent of area tempestas,” exhibiting increased hemodynamic responses in relation to interictal epileptiform discharges. Based on the reported Talairach coordinates, we previously hypothesized that this area corresponds to the ventral claustrum (Meletti et al., 2015). To further support our hypothesis, we show EEG-fMRI results from this and subsequent studies that attribute interictal discharge-related hemodynamic responses to ‘area tempestas’ that correspond to the location of the ventral claustrum (Figure 1D).

Interestingly, Laufs et al. also found reduced benzodiazepine-GABA_A_ receptor binding complex expression in ‘area tempestas,’ as measured by flumazenil positron emission tomography, in patients experiencing more frequent seizures. The claustrum notably harbors a significant population of GABAergic interneurons, which are influenced by anesthetic agents that interact with benzodiazepine GABA_A_ receptor binding complexes. Consequently, reductions observed in the expression of these complexes may in fact occur within the claustrum. This reduction may also be linked to the decreased effectiveness of benzodiazepines observed in cases of refractory SE (Singh et al., 2014; Borroto-Escuela and Fuxe, 2020; Kim et al., 2020; Luo et al., 2023).

In line with these results, in Figure 1C we illustrate a putative seizure generalization network from limbic-associated brain structures to motor-related cortical areas via the ventral claustrum. We hypothesize that seizures arising from temporal lobe structures can generalize broadly across ipsi- and contralateral neocortices (e.g., generalized tonic-clonic seizures) through ventral claustrum projections. Considering this subcortical generalization network, we further speculate that anterior temporal lobectomy and other temporal lobe resection techniques utilized in epilepsy could, in some cases, resect the ventral portion of the claustrum or transect limbic fibers to this subregion (Feindel et al., 2009; Borger et al., 2021; Dalio et al., 2022). The incidence, benefits, and/or altered outcomes of these surgical possibilities are unknown. However, a recent case study provides strong evidence that not fully resecting ‘area tempestas’ may cause seizure recurrence following temporal lobectomy (Garganis et al., 2013; Figure 1D).

## 6 Is the claustrum a suitable therapeutic target in epilepsy?

Our perspective on available evidence implicates the ventral claustrum as a key node within a dysfunctional epileptic network. To review, four key pieces of evidence from clinical and animal studies point to the claustrum as a useful target for therapeutic development in epilepsy: (1) Neuroimaging data that show increased activation in a region that stereotactically corresponds to the ventral claustrum; (2) Histopathological data in both animals and humans that reveal neuronal cell death and cellular alterations in the ventral claustrum after uncontrolled seizures; (3) Electrical kindling data revealing that the ventral claustrum has a low threshold and high susceptibility to seizure induction, and lesions to this region can profoundly mitigate or block seizure generalization; (4) Seizure-induced disruptions to claustro-cortico-thalamic interactions that constitute brain wide ICNs could impair consciousness during certain seizure subtypes. Considering this evidence, we conclude that the ventral claustrum represents a viable target in correcting a dysfunctional epileptic network. Below we review several therapeutic possibilities.

Targeting endogenous opioids has recently gained attention as a promising therapy to treat temporal lobe epilepsy (Zangrandi and Schwarzer, 2022; Lankhuijzen and Ridler, 2024). As described earlier, the claustrum has a high density of KORs, presenting a unique opportunity to target this brain region with KOR agonists to reduce neuronal excitability, especially during SE (Kumar et al., 2023). Therefore, the use of KOR agonists as anticonvulsants, specifically for refractory SE, should be explored further. More work is also needed to explore how the use of benzodiazepine and non-benzodiazepine GABA_A_ modulators can be used to selectively target claustrum interneurons during seizures.

We previously discussed data hinting at the possibility that resecting the ventral claustrum could, theoretically, provide benefit in patients with generalized seizures that arise from temporal lobe structures (Feindel et al., 2009; Borger et al., 2021; Dalio et al., 2022). However, this viewpoint is highly speculative and requires formal investigation to support or refute. Magnetic resonance-guided focused ultrasound to selectively ablate the ventral claustrum may provide a starting point to test this hypothesis (Ranjan et al., 2019). Long-standing neuromodulation techniques can also be used to target the claustrum, especially with closed-loop, state-dependent stimulation (Wong et al., 2021; Watson and Kopell, 2022). It is conceivable that claustrum electrical stimulation may help correct an aberrant epilepsy network or prevent seizure generalization, but confounding variables such as the alteration of consciousness seen in the N-of-1 study discussed (Koubeissi et al., 2014), and the possibility of off-target white matter stimulation effects may make this modality less favorable (Kurada et al., 2019). Owing to the claustrum’s unique anatomy, more advanced cell- and pathway-specific neuromodulation techniques to effectively target this structure are warranted (Watson et al., 2021b).

An emerging tool that could selectively target the claustrum to treat epilepsy is gene therapy (Shaimardanova et al., 2022; Boileau et al., 2023; Miyakawa et al., 2023). Diffuse or more targeted use of viral promoters (e.g., adeno-associated viruses, AAVs) are being used to restrict vector expression to select populations of neuronal subtypes. Gene therapy would address the issue of non-specific neuromodulation and the systemic targeting of many antiseizure medications. For example, selectively attenuating glutamatergic projection neurons in the ventral claustrum through gene therapy may prevent seizure generalization or impaired awareness as previously discussed. Even developmental and epileptic encephalopathies such as Dravet syndrome may benefit from selective targeting of Nav1.1 parvalbumin neurons in the claustrum (Vormstein-Schneider et al., 2020; Niibori et al., 2023).

As we contemplate the therapeutic potential of targeting the claustrum, the prospect of correcting a dysfunctional epileptic network becomes both promising and challenging. This perspective article opens new avenues for understanding the intricate interplay between the claustrum and limbic brain structures, providing a foundation for future research and potential breakthroughs in epilepsy therapeutics.

## Data Availability

The original contributions presented in the study are included in the article/Supplementary material, further inquiries can be directed to the corresponding author.
